# Comparison of Arrhythmogenicity and Proinflammatory Activity Induced by Intramyocardial or Epicardial Myoblast Sheet Delivery in a Rat Model of Ischemic Heart Failure

**DOI:** 10.1371/journal.pone.0123963

**Published:** 2015-04-10

**Authors:** Tommi Pätilä, Shigeru Miyagawa, Yukiko Imanishi, Satsuki Fukushima, Antti Siltanen, Eero Mervaala, Esko Kankuri, Ari Harjula, Yoshiki Sawa

**Affiliations:** 1 Department of Cardiothoracic Surgery, Osaka University Graduate School of Medicine, Osaka, Japan; 2 Department of Cardiothoracic Surgery, University of Helsinki and Helsinki University Hospital, Helsinki, Finland; 3 Pharmacology, University of Helsinki, Helsinki, Finland; 4 Department of Pediatric Cardiac Surgery, Hospital for Children and Adolescents, University of Helsinki, Helsinki, Finland; Tokai University, JAPAN

## Abstract

Although cell therapy of the failing heart by intramyocardial injections of myoblasts to results in regenerative benefit, it has also been associated with undesired and prospectively fatal arrhythmias. We hypothesized that intramyocardial injections of myoblasts could enhance inflammatory reactivity and facilitate electrical cardiac abnormalities that can be reduced by epicardial myoblast sheet delivery. In a rat model of ischemic heart failure, myoblast therapy either by intramyocardial injections or epicardial cell sheets was given 2 weeks after occlusion of the coronary artery. Ventricular premature contractions (VPCs) were assessed, using an implanted three-lead electrocardiograph at 1, 7, and 14 days after therapy, and 16-point epicardial electropotential mapping (EEPM) was used to evaluate ventricular arrhythmogenicity under isoproterenol stress. Cardiac functioning was assessed by echocardiography. Both transplantation groups showed therapeutic benefit over sham therapy. However, VPCs were more frequent in the Injection group on day 1 and day 14 after therapy than in animals receiving epicardial or sham therapy (p < 0.05 and p < 0.01, respectively). EEPM under isoproterenol stress showed macroreentry at the infarct border area, leading to ventricular tachycardias in the Injection group, but not in the myoblast sheet- or sham-treated groups (p = 0.045). Both transplantation types modified the myocardial cytokine expression profile. In animals receiving epicardial myoblast therapy, selective reductions in the expressions of interferon gamma, interleukin (IL)-1β and IL12 were observed, accompanied by reduced infiltration of inflammatory CD11b- and CD68-positive leukocytes, compared with animals receiving myoblasts as intramyocardial injections. Intramyocardial myoblast delivery was associated with enhanced inflammatory and immunomodulatory reactivity and increased frequency of VPCs. In comparison to intramyocardial injection, the epicardial route may serve as the preferred method of skeletal myoblast transplantation to treat heart failure.

## Introduction

Cell therapy of ischemic heart failure with skeletal myoblasts initiated the first hype in cardiac cell transplantation more than a decade ago [1–4]. Transplanted myoblasts populated the heart and reversed ventricular remodeling through secretion of paracrine mediators [5]. In addition, isolation and expansion of autologous myoblasts did not involve complicated laboratory procedures, genetic manipulation, or allogeneic material, allowing swift application in the clinical arena. Since then, several studies—including large-scale randomized clinical trials—have implicated a limited therapeutic efficacy and possibly fatal arrhythmogenicity for myoblast cell therapy [6–11]. These undesired effects were explained, at least in part, by inconsistencies in cell transplantation protocols (number of myoblasts transplanted, methods of myoblast delivery into the heart) or target heart pathology that could fundamentally affect the behavior of the transplanted myoblasts.

The route of delivery plays an important role in the arrhythmogenicity of cell transplantation in chronic heart failure [12]. Cell delivery by intramyocardial injections causes formation of heterogenic cell clusters that consist of the transplanted cells and infiltrated host cells, leading to formation of potential areas of electrical reentry that may cause ventricular tachyarrhythmias [13]. On the other hand, epicardial transplantation of scaffold-free cell sheets on the surface of the heart promotes survival of the transplanted cells and enhances the therapeutic effects of myoblast delivery [4]. A recent report by Narita *et al*. demonstrated a low level of arrhythmogenicity for cell-sheet transplantation [14]. We hypothesized that selective modification of myocardial inflammatory reactivity by direct cell injections into myocardial tissue may be associated with ventricular arrhythmias. Moreover, we investigated whether such inflammatory reactivity and associated arrhythmias could be avoided by epicardial myoblast sheet transplantation without compromising therapeutic efficacy.

## Materials and Methods

All animals were maintained and study procedures performed in accordance with the "Principles of Laboratory Animal Care" formatted by the National Society for Medical Research. All animal experiments were approved by the Local Ethics Committee of Osaka University Hospital. All procedures and measurements were performed blinded to the treatment methods when applicable.

### Primary skeletal myoblasts and myoblast sheets

Primary skeletal myoblasts were isolated from the tibial anterior muscles of 3-week-old male Lewis rats (n = 10, weight 100–150 g), as described previously [2]. Briefly, the muscles were excised and washed with phosphate-buffered saline (PBS). After meticulous removal of nonmuscle tissues, the muscles were weighed, minced, and enzymatically digested with collagenase (Gibco BRL, Rockville, MD, USA), 5 mg/mL at 37°C for 40 min. The cells were collected and resuspended in Dulbecco’s modified Eagle’s medium (DMEM) (Gibco BRL) with 20% fetal bovine serum (FBS) and 1% penicillin-streptomycin (Gibco BRL). The cells were plated in 100-mm collagen-coated culture dishes (Iwaki Co. Ltd., Tokyo, Japan) and cultured at 37°C in a humidified atmosphere containing 5% CO_2_. Poly-N-isopropylacrylamide (PIPAAm) thermoresponsive polymer-coated culture dishes were used to construct the myoblast sheets [15]. The surfaces of these dishes are hydrophobic at an incubator temperature of 37°C, but become hydrophilic at temperatures less than 20°C. A total of 9 × 10^6^ cells in suspension were plated on the PIPAAm cell-culture dish and incubated for 1–2 days, as outlined above. Thereafter, the cell sheets spontaneously detached from these dishes at 20°C in 1 h. For the injections, a myoblast single-cell suspension of 9 x 10^6^ cells was used.

### Model of ischemic cardiomyopathy and skeletal myoblast transplantation

Female Lewis rats 8 weeks of age (175–225g) underwent permanent ligation of the left anterior descending (LAD) artery at the level of the left atrial appendage through the 4^th^ intercostal space (n = 70) [16]. Two weeks after the LAD ligation, a rethoracotomy through the 5^th^ intercostal space was performed for myoblast transplantation. The rats were randomized into three groups: 1) direct intramyocardial injection of 9 x 10^6^ cells in 100 μl PBS, using a 31-gauge needle (Injection group, n = 17), 2) epicardial application of a myoblast sheet, consisting of 9 x 10^6^ cells, on the infarct area (Sheet group, n = 18), or 3) sham operation (Control group, n = 19). The sham-operated rats underwent induction of infarction by LAD ligation and a rethoracotomy after 2 weeks without cell transplantation.

### Echocardiography

**Fig 1:**
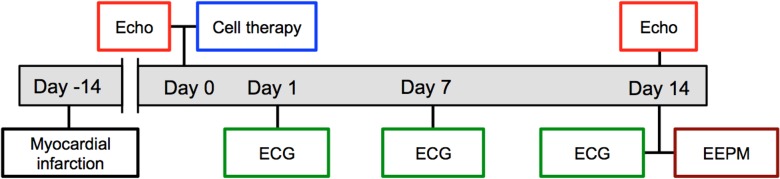
Study protocol timeline. Myoblast cell therapy as intramyocardial injections or epicardial sheets was administered 2 weeks after induction of heart failure and ischemia by ligation of the left anterior coronary artery (Myocardial infarction). Electrocardiography (Holter) was monitored constantly, using implanted Holter monitors, and specific recordings were made on days 1, 7, and 14 after cell therapy administration. Echocardiography (Echo), as measured first before and then 2 weeks after myoblast transplantation, was used to evaluate therapy efficacy. Epicardial electropotential mapping (EEPM) was performed 14 days after therapy administration.

Fig 1 illustrates the experimental protocol. Echocardiography (ECG) was performed before the LAD ligation as well as before and 2 weeks after myoblast transplantation. The rats were anesthetized by 2% isoflurane inhalation for transthoracic ECG, and the end-diastolic and end-systolic diameters (LVEDD and LVESD, respectively) of the left ventricles (LVs) were measured at the level of the papillary muscles, using the M-mode. The two-dimensional mode was used to assess the LV ejection fraction (LVEF).

### Telemetric electrocardiography

Each animal was implanted with a three-lead electrocardiographic device (Unique Medical Co. Ltd, Tokyo, Japan) into its back before myoblast transplantation at 2 weeks after LAD ligation. The electrodes were placed at shoulder level and at the midline of the nape. A 6-h electrocardiographic assessment was performed on all animals on days 1, 7, and 14 after treatment to record the ECG and signal intensification, which were analyzed by Unique Medical Acquisition Software (Unique Medical Co. Ltd.). The number of ventricular premature contractions (VPCs) was calculated as the percentage of all ventricular beats. Multiform consecutive VPCs and ventricular tachycardias (VTs) were quantified as the number of events during a 24-h period.

### Epicardial electropotential mapping under isoproterenol stress

After the 14-day follow-up (Fig 1), 16-point epicardial electropotential mapping (EEPM) was performed with an epicardial multielectrode probe (Unique Medical Co. Ltd.). This probe consists of 16 needle electrodes, molded in a 5 x 5-mm silicone plate in a quadrangular fashion. Each electrode’s electric potential was recorded at 2.0-ms intervals. These recordings were performed at four points in the infarct border area before and after administration of the proarrhythmic drug, isoproterenol (0.1 μmol/L, Sigma-Aldrich, Tokyo, Japan) by transapical bolus injection.

### Histological studies

After the 14-day follow-up, following ECG and electrocardiography assessments, the rats were euthanized under anesthesia by potassium chloride injection through the apex of the heart. The hearts were harvested and the LVs sliced into four pieces along the short axis. The specimens were fixed with 4% formaldehyde and embedded in paraffin. Sections (10 μm thick) were examined by immunofluorescence for leukocyte/monocyte infiltration by cluster of differentiation (CD)11b (Abcam plc, Cambridge, UK) expression with 4',6-diamidino-2-phenylindole (DAPI, Sigma-Aldrich) counterstaining. Monocyte/macrophage infiltration was further confirmed with immunohistochemical staining of CD68. Antigen retrieval was done at 95°C for 1 h in 10 mM citrate buffer and endogenous peroxidase was blocked by 0.5% H_2_O_2_-treatment for 30 min. The sections were then blocked in 10% normal goat serum (Vector Laboratories, Peterborough, UK), followed by incubation with primary antibody against CD68 (Abcam, 1:100 dilution) and biotinylated secondary antimouse antibody (Vector Laboratories). The sections were then further treated, using the Vectastain Elite ABC kit followed by the 3-amino-9-ethylcarbazole (AEC) Peroxidase Substrate Kit (Vector Laboratories). Prior to mounting, the sections were counterstained with eosin. Six images per myocardial section were used (20x magnification) to calculate the mean values of the positively stained area. The images were analyzed, using ImageJ software (imagej.nih.gov/ij/).

### Evaluation of myocardial gene expression by real-time PCR

Immediately after harvesting, the midventricle infarct border area was cut and immersed in RNAlater RNA-Stabilization Reagent (Qiagen, Hilden, Germany) to stabilize the RNA (n = 18, six animals from each group). The total RNA was then isolated (RNeasy Mini Kit, Qiagen) and purified (RNase-Free DNase Kit, Qiagen). Reverse transcription was executed with 1 μg of total RNA (Omniscript Reverse Transcriptase, Qiagen). The sets of primers and a TaqMan probe, which was labeled with a reporter dye, fluorescein amidite (FAM) at the 5′-end and a quencher dye, carboxytetramethylrhodamine (TAMRA) at the 3′-end, were designed with Primer Express software (Life Technologies, ThermoFisher, Waltham, MA, USA). The real-time polymerase chain reaction (rtPCR) was carried out, using the ABI PRISM 7700 Sequence Detection System with TaqMan Universal PCR Master Mix (Applied Biosystems). Absolute quantification was used to determine the number of fragments (copy number), using external standards. A PCR product containing the target sequence was used as the standard and was evaluated at seven different copy numbers. Relative quantification was calculated by the ratio between the amount of target and glyceraldehyde 3-phosphate dehydrogenase (GAPDH) within the same sample. The sequences for the primers and probes used in this study are listed in S1 Table.

### Statistical analysis

All values are expressed as mean ± standard error of the mean (SEM). The differences between groups were compared, using one-way analysis of variance (ANOVA) and Newman-Keul’s posttest with multiple comparisons. Contingency tables were analyzed with Fisher’s test (mortality and VT’s). Pairwise comparisons were performed using student’s t-test. Statistical analyses were performed with Graph Pad Prism 4.0 (GraphPad Software Inc., San Diego, CA, USA). A value of p < 0.05 was considered significant.

## Results

Mortality following the LAD ligation was 14% (10/70). All mortality occurred during or soon after the surgical procedure, due to surgical stress. Prior to randomization, six rats were excluded from the study, due to the lack of infarction in the pretreatment ECG. Mortality following the surgery for transplantation therapy was 14% (3/21) in the Sheet group, 19% (4/21) in the Injection group, and 14% (3/22) in the sham rethoracotomy group, with no statistical differences between the groups.

### Therapeutic efficacy of myoblast transplantation

**Fig 2:**
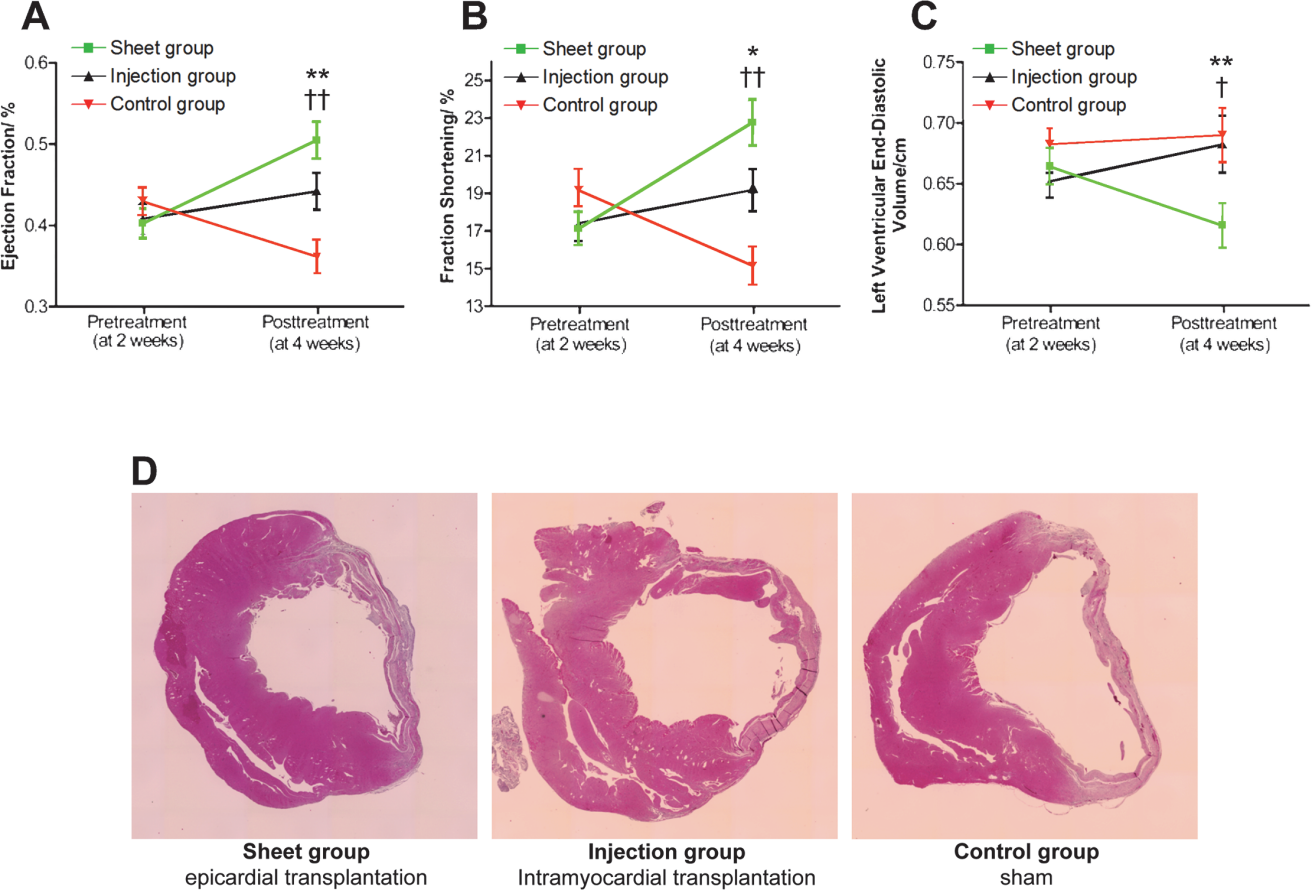
Panel A: Epicardial transplantation of myoblast sheets (Sheet group, n = 18) significantly improved the left ventricular ejection fraction (EF). In sham-treated animals (Control group, n = 19), EF decreased markedly during the 2-week follow-up. This decrease in EF was attenuated by myoblast intramyocardial injection therapy (Injection group, n = 17). Panel B: Significant improvement in fractional shortening (FS) was observed in the Sheet group. FS significantly deteriorated in the Control group, while in the Injection group this decrease was attenuated. Panel C: As a measure of left ventricular remodeling, the left ventricular end-diastolic diameter (LVEDD) decreased in the Sheet group and increased in the Control group. In the Injection group, the LVEDD remained similar to the baseline value. Panel D: Representative hematoxylin-eosin-stained paraffin-embedded sections of the heart on the midventricular short axis from each group. The myocardium in the Sheet group demonstrated less remodeling of the left ventricle than the Control group. A similar, but less pronounced, effect on remodeling was evident in the Injection group. * p < 0.05, ** p < 0.01 Sheet group vs. Control group; † p < 0.05, †† p < 0.01 Sheet group vs. Injection group.

To evaluate the regenerative therapeutic efficacy of the treatment modalities, LVEF, LVEDD, and LVESD were assessed before and 2 weeks after the treatment, using transthoracic ECG (Fig 2). LVEF was significantly increased in the Sheet (40 ± 5–50 ± 7%) and the Injection (41 ± 6–44 ± 7%) groups at follow-up, but was decreased in the Sham group (43 ± 5–36 ± 7%) (Fig 2A). A similar result was found for fractional shortening (FS) (Fig 2B). LVEDD increased in the Control group (6.1 ± 0.9–6.9 ± 0.7 mm), while this increase was not observed in the Sheet (6.5 ± 0.4–6.8 ± 0.7 mm) or Injection (6.5 ± 0.4–6.8 ± 0.7) groups (Fig 2C). Fig 2D shows representative hematoxylin-eosin-stained images of the myocardium at the 14-day follow-up from each group to demonstrate the effect of myoblast transplantation on remodeling of the LV.

### Reduced spontaneous arrhythmias following epicardial myoblast sheet transplantation

**Fig 3:**
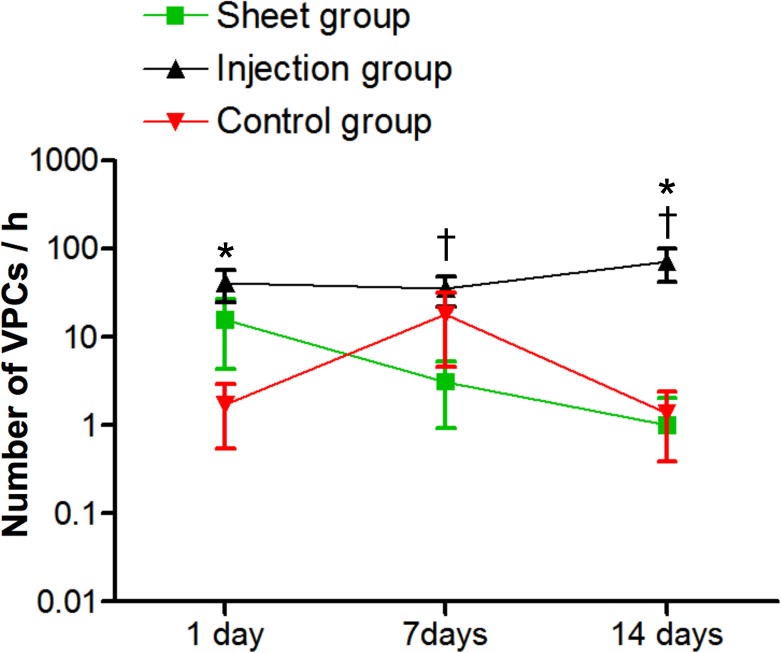
Ventricular premature contractions (VPCs) after epicardial myoblast sheet transplantation (Sheet group, n = 18) or intramyocardial myoblast injections (Injection group, n = 17). The Control group (n = 19) received, instead of myoblast therapy, a sham operation 2 weeks after myocardial infarction. Electrocardiography was monitored continuously. The number of VPCs was significantly higher in the Injection group on days 1 (p < 0.05) and 14 (p < 0.01) after myoblast transplantation than in the Control group (*) and significantly higher on days 7 (p < 0.05) and 14 (p < 0.01) than in the Sheet group (†). There was no ventricular tachycardia recorded in any of the animals.

A 6-h continuous ECG monitoring was performed, using telemetry on days 1, 7, and 14 after the myoblast transplantations. The Injection group showed more VPCs than the Control group on days 1 and 14, while the Injection group showed more VPCs than the Sheet group on days 7 and 14 (Fig 3). There was no significant difference between the Sheet group and the Control group: (Sheet: 15.6 ± 11.2 on day 1, 3.1 ± 2.2 on day 7, and 1.0 ± 1.0 on day 14; Injection: 41.3 ± 16.4 on day 1, 35.2 ± 13.3 on day 7, and 71.5 ± 29.8 on day 14; Control: 1.7 ± 1.2 on day 1, 18.0 ± 13.5 on day 7, and 1.4 ± 1.0 on day 14. No VTs were detected in any of the rats during the Holter recordings.

### Intramyocardial myoblast injection-induced formation of electrical reentry

**Fig 4:**
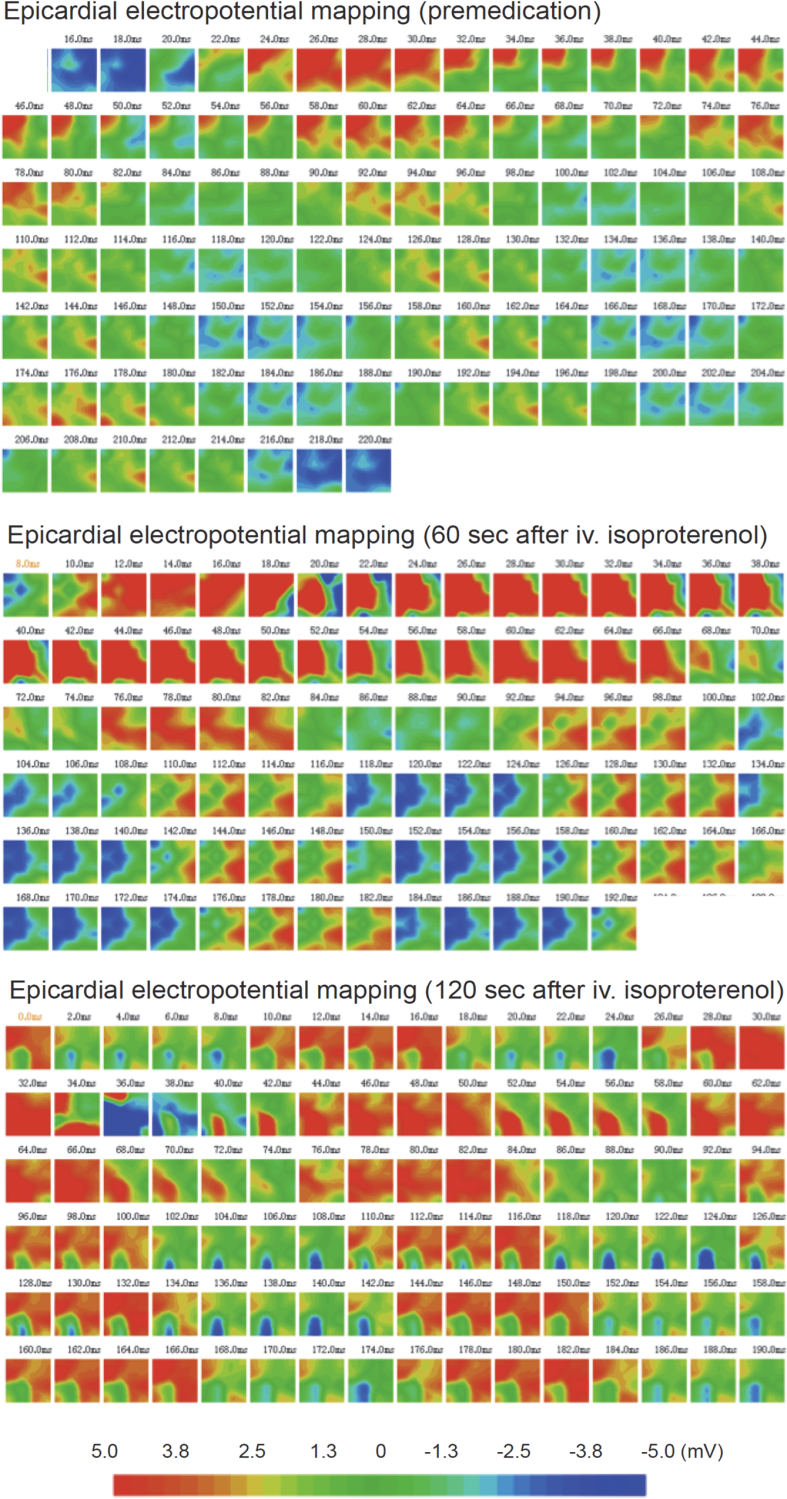
Electropotential mapping was performed epicardially with a 16-needle probe at 2.0-ms intervals. In the premedication maps, one cardiac R-R-cycle is shown (images from 18 ms to 218 ms) at a normal rat heart rate (300 beats per minute, bpm). An area of reentry can be seen as red coloration in the middle of the border area measured. Sixty seconds after a bolus injection of isoproterenol, the reentry area was observed, while 120 s after the isoproterenol bolus, a full ventricular tachycardia has developed, with a heart rate of approximately 6000 bpm.

EEPM showed no significant differences in QRS wavelength, activation/repolarization intervals, or refractory times among the groups. However, two rats of the Injection group showed an area of macroreentry at the infarct border where the myoblasts were injected (Fig 4). In both of these rats, isoproterenol administration caused a VT that developed from the site of the reentry. In two other rats of the Injection group, isoproterenol administration induced VT without clear detection of an area of reentry. The Sheet or Control groups did not show any macroreentry areas or induce VTs by isoproterenol administration (p = 0.045).

### Leukocyte infiltration and inflammatory gene expression in the myocardium

**Fig 5:**
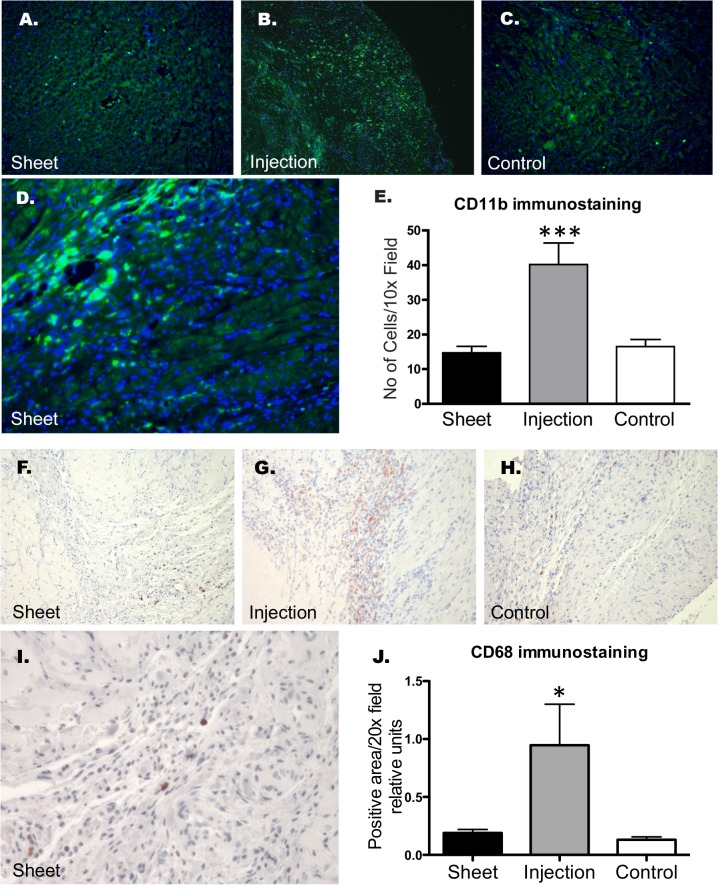
Panels A-C: Representative immunofluorescence images of CD11b-expression, with DAPI counterstaining of the nuclei of the myocardium 2 weeks after myoblast cell therapy (4 weeks after myocardial infarction). Infiltration of CD11b-positive cells by epicardial transplantation (panel A) was similar to that of the Control group (panel C). Panel D: Clusters of CD11b-positive inflammatory cells can be seen in the myocardium of the Injection group at a higher magnification. Panel E shows densitometry quantitation of CD11b-expression in the various groups (n = 6). In the group receiving intramyocardial injections, an increased leukocyte infiltrate was evident in comparison to the Control or Sheet groups (*** p < 0.001 vs. Injection group). Panels F-I show CD68-staining similar to that of CD11b. Quantitation of the CD68-positive area showed increased CD68-positive cell infiltration in the Injection group in comparison to the Sheet and Control groups (panel J, * p < 0.05 for both comparisons).

Immunofluorescent staining for CD11b (p < 0.001) and immuhistochemistry for CD68 (p < 0.05, Fig 5) detected less macrophage accumulation in the infarct border areas of the Sheet and Control groups than in that of the Injection group at the 14-day follow-up.

**Fig 6:**
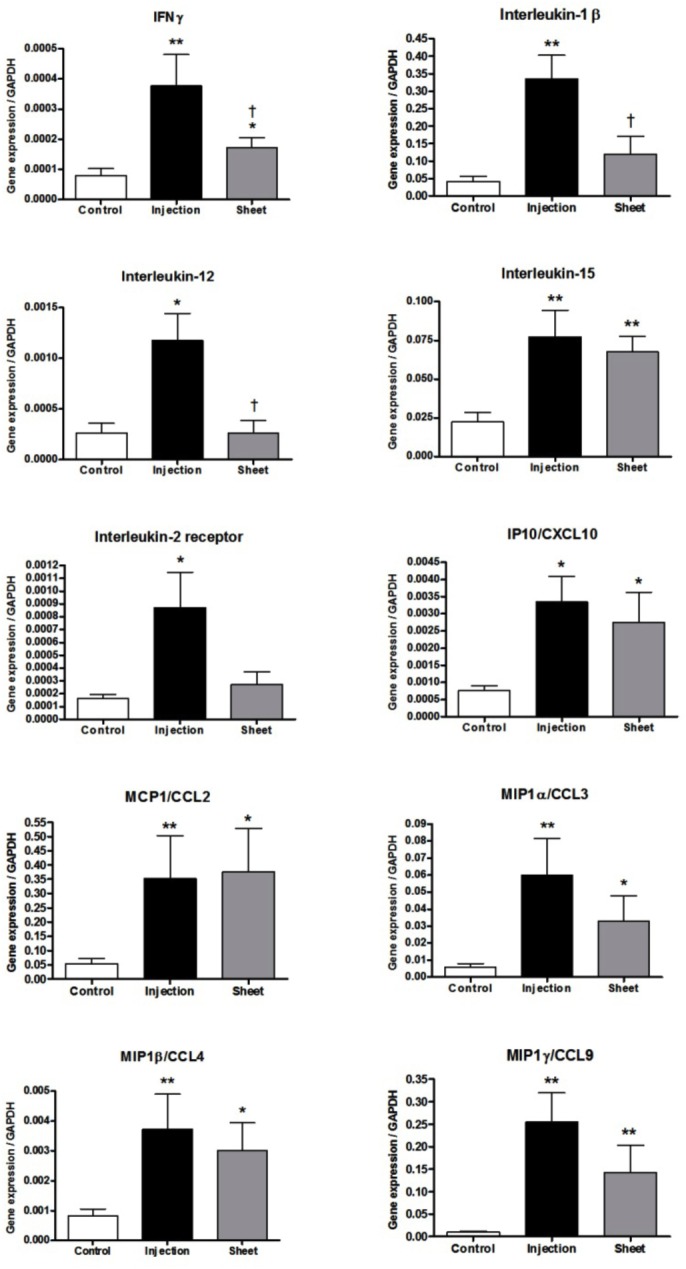
Myocardial gene expressions of inflammatory genes in the sham-treated (Control group, n = 6) and in the groups receiving myoblast cell therapy as intramyocardial injections (Injection group, n = 6) or epicardial cell sheets (Sheet group, n = 6). Data are expressed as mean ± SEM. * p < 0.05, ** p < 0.01 compared with the Control group. † p < 0.05 compared with the Injection group.

Inflammatory gene expression in the myocardium was evaluated, using rtPCR. Fig 6 demonstrates the expression patterns of interferon (IFN)γ, IL1β, IL12, IL15, IL2R, inducible protein 10/chemokine (C-X-C motif) ligand 10 (IP10/CXCL10), monocyte chemoattractant protein 1/chemokine (C-C motif) ligand 2 (MCP1/CCL2), macrophage inflammatory protein 1α/chemokine (C-C motif) ligand 3 (MIP1α/CCL3), MIP1β/CCL4, and MIP1γ/CCL9, which differed statistically between animals in the Injection group and those in the Control group. The expressions of IL6 and IL10 in the myocardium remained low in all groups, and there were no differences between the groups. The expression of connexin 43 (CNX43) was not significantly different statistically among the groups. Interestingly, the selective expressions of IFNγ, IL1β, and IL12 genes were significantly greater in the Injection group than in the Sheet group. The expression levels of IL1β and IL12 in the Injection group did not differ from the relatively low expression levels observed in the Control group.

## Discussion

Using a rat model of ischemic heart failure, we show here that epicardial transplantation of myoblast sheets prevents the undesired development of spontaneous ventricular tachyarrhythmias associated with cell delivery by intramyocardial injections. Reentry tachycardias provoked by an adrenergic isoproterenol challenge were observed only in the group treated with skeletal myoblast injections. Moreover, myoblast injections—but not epicardial deposition—increased the myocardial inflammatory infiltrate (as evidenced by the number of CD11b- and CD68-positive cells) and increased the gene expression of IL1β, IL12, and IFNγ proinflammatory cytokines. Therapy of ischemic heart failure by epicardial myoblast sheets improved cardiac functioning, with superior efficacy over myoblast therapy by intramyocardial injections.

Intramyocardial myoblast delivery generates cell clusters of transplanted cells, results in accumulation of host-derived inflammatory cells, and induces ventricular tachyarrhythmias [8,17]. The role played by the inflammatory process in these myocardial electrical abnormalities has remained unclear. Myocardial infarction is closely linked to inflammation and increased production of inflammatory cytokines [18]. Intramyocardial injections may induce host myocardial injury and/or death of injected cells, processes that signal for initiation and acceleration of the inflammatory process in the longer run. Our results demonstrate that increased or sustained inflammatory leukocyte infiltration and selective expression of the proinflammatory cytokines IL1β, IL12, and IFNγ are linked to myoblast intramyocardial delivery and are associated with the VPCs induced by this type of therapy administration. Of note, isoproterenol-stimulated electrical macroreentry, as visualized here using EEPM in two myoblast-injected rats, suggests that the inflammatory infiltrate or sustained production of IL1β/IL12/IFNγ positively influences the process of macroreentry. By contrast, epicardial myoblast-sheet implantation did not induce accumulation of macrophages and, importantly, neither produced an electrical macroreentry area nor enhanced the ventricular tachyarrhythmic potential.

To achieve clinical relevance with timing of cell transplantation, myoblast therapies were administered 14 days after the induction of myocardial infarction. In this model, therapeutic benefit was more evident following epicardial myoblast sheet implantation than direct myoblast injection. Since the expression profiles of several cytokines were similar between the Injection and Sheet groups, the difference in therapeutic effect likely resulted from differential survival of myoblasts and the magnitude of acute inflammation postimplantation. Since epicardial cell transplantation increases cell survival in comparison to cell injections [19], its greater therapeutic effects can be mediated by increased amounts of myoblast-derived cardioprotective paracrine factors. Although direct myocardial cell injection up-regulates cardioprotective factors in the myocardium, it also induces up-regulation of the proinflammatory cytokines, including IL1β, that adverse affect the contractile function of cardiomyocytes [20] or serve to exacerbate myocardial fibrosis [21]. Moreover, myoblast-sheet transplantation results in higher myocardial graft retention and more even graft distribution at the site of transplantation, as opposed to injection-associated formation of cell clusters and cell localizations in the lungs [14].

As outlined above, the cytokines observed here as being selectively induced by intramyocardial myoblast injections were IL1β, IL12, and IFNγ. Taken together with increased CD11b-/CD68-positive leukocyte infiltration, a hypothesis of macrophage subtype modulation by these therapy types can be postulated. Increased production of IL12 from dendritic cells and monocytes in response to intracardiac myoblast delivery by injections could stimulate helper 1 T (T_H_1) and natural killer (NK) cells to produce IFNγ, which in turn could shift macrophage polarity towards an IL1-producing proinflammatory M1 subtype [22]. Predominance of this subtype, implicated in the acute phase of injury after myocardial infarction [23,24] could then limit the therapeutic potential of myoblasts. In turn, attenuating the host myocardial IL12 response by epicardial myoblast delivery could drive the myocardial cytokine profile into one serving a more regenerative and restorative function [24]. This would be suggested by the dual nature of MCP1 in the heart [25,26].

IL1 has been directly implicated in heart failure pathogenesis [27,28], negative modulation of cardiomyocyte contractility, and electrical conduction [29,30]. This cytokine is produced in response to tissue damage, primarily by tissue-infiltrating monocytes [30]. Importantly, suppression of myocardial IL1 signaling can improve the survival of grafted myoblasts, suggesting an essential role for suppression of the IL1 pathway in myoblast therapy [31]. Therefore, selective modulation of this cytokine, as seen here by choice of transplantation technique, may help to attenuate its adverse effects on myocardial functioning in cell transplantation. The role of IL1 in adverse remodeling and in both direct and indirect (e.g. pathological cardiac morphology, stimulation of immunological and inflammatory signaling) modification of cardiac electrophysiological conduction circuitry deserves further investigation.

The selective induction of IL12 by intramyocardial injections of myoblasts, as evidenced here, should be evaluated further. IL12 is produced mainly by cells of the immune system, such as monocytes and dendritic cells [32], and it regulates the functioning of NK cells and T-cells [32]. Increased IL12 after intramyocardial myoblast delivery, but not after epicardial cell deposition, may reflect a selective innate host response against the injected cells. In fact, the blockade of IL12 attenuates chronic rejection in cardiac transplantation [33], suggesting that the IL12 host response may accentuate myocyte death by immunological mechanisms in intramyocardial delivery. Although previous studies have reported little of the immune response-related inflammation we showed in the model used here, our study may have been limited by the hostimmune response, due to gender-mismatched transplantation [34,35]. However, the lack of an IL12 response against epicardially transplanted cells, as we observed here, may enhance survival of the transplanted cells and favorably modulate the myocardial inflammatory signaling networks and immunological injury [36].

We were unable to repeat the finding of Coppen *et al*. [37] that secreted IL1β from the surviving skeletal myoblasts globally down-regulates myocardial CNX43 expression and consequently induces ventricular tachyarrhythmia following transplantation of skeletal myoblasts in a chronic heart failure model. In concert with that study, however, we found up-regulation of IL1β—but not CNX43 as stated above—after myoblast injection, but not after cell-sheet implantation. This may have been due to differences in experimental models, such as magnitude of the myocardial infarction, timing of the treatment, or rat strain. As a limitation of our study, we did not measure direct cell-cell coupling between native cardiomyocytes and transplanted myoblasts.

This study is further limited by the fact that we compared two administration routes and did not include various other means of cell delivery, such as intracoronary infusion. It needs to be pointed out that—in concert with our results on the therapeutic inferiority of intramyocardial delivery—Li *et al*. (2011) demonstrated that intracoronary cell delivery also resulted in greater therapeutic effect and more uniform cell distribution than intramyocardial injections [38]. Further studies are warranted to directly compare the effects of cell-sheet and intracoronary infusion techniques. Moreover, a combination approach with cell sheets first transplanted epicardially during bypass surgery, followed by cells delivered periodically using a transcatheter infusion approach, possibly even in a repeated manner over time, could be envisioned as providing synergistic and longer-lasting effects. Another limitation of our study is the lack of fibrosis quantitation. Memon *et al*. previously showed that the epicardial delivery route results in reduced fibrosis in contrast to intramyocardial injections [2]. The reduced inflammatory response associated with the epicardial delivery route may contribute to the antifibrotic effect reported.

In summary, our results demonstrate that injection of myoblasts into the LV after resolution of the acute first phase of inflammation induced by myocardial ischemia incites an inflammatory reaction characterized by CD11b-/CD68-positive leukocyte infiltration that is not observed in epicardial delivery of myoblasts. Epicardial transplantation also significantly reduced both VPCs and the expressions of IL1β, IL12, and IFNγ in the myocardium. As a preliminary finding warranting further investigation, this result suggests a role for these cytokines and putative modulation of leukocyte trafficking in the induction of arrhythmogenicity during cell transplantation. We demonstrate here that epicardially transplanted myoblast sheets lack the arrhythmogenic and inflammatory activity that is induced when myoblasts are delivered intramyocardially. Our results thus indicate that epicardial delivery of myoblasts has a positive safety profile and that this method thus has ideal properties as a cellular therapy modality for patients with ischemic heart failure.

## Supporting Information

S1 Table Forward and reverse primers and probes used in this study to detect expression of the genes listed in the myocardial samples by real-time PCR, as detailed in the Materials and Methods section. (DOC)
