# Supplementary material for: Comparison of Arrhythmogenicity and Proinflammatory Activity Induced by Intramyocardial or Epicardial Myoblast Sheet Delivery in a Rat Model of Ischemic Heart Failure
Source: PLoS One. 2015 Apr 10;10(4):e0123963. doi: 10.1371/journal.pone.0123963 (PMC4393220; doi:10.1371/journal.pone.0123963)
Supplement: S1 Table — (DOC) [file pone.0123963.s001.doc]

**Primers and probes**

| Recombinant | F-primer | R-primer | Probe |  |  |  |
| --- | --- | --- | --- | --- | --- | --- |
| GAPDH | CCA TCA CTG CCA CTC AGA AGA C | TCA TAC TTG GCA GGT TTC TCC A | CGTGTTCCTACCCCCAATGTATCCGT | | | |
| MCP-1 | TGTCTCAGCCAGATGCAGTTAA | GCACAGATCTCTCTCTTGAGCTTG | TGATCCCAATGAGTCGGCTGGAGAACTA | | | |
| IFN-gamma | ATCGAATCGCACCTGATCACTA | TTCTTATTGGCACACTCTCTACCC | AACAACCCACAGATCCAGCACAAAGC | | | |
| IP10 | CAGAAGCACCATGAACCCAAG | TCAACATGCGGACAGGATAGAC | ATCGACTTCCATGAACAGCCGCTGA | | | |
| Connexin43 | TGGTGACAGAAACAATTCCTCG | GGCTAATGGCTGGAGTTCATGT | CGAACTACAGCGCAGAGCAAAATCGCAT | | | |
| IL2 receptor | CCCTCAGGTGTTTCTTGAGCTT | CTTTCCCAGAGAGTGAGGCTTC | TGGCCACTGCTACCTGATACTCCTTTGTGA | | | |
| IL10 | ATAACTGCACCCACTTCCCAGT | GGAGAAATCGATGACAGCGTC | ACCATGGCCCAGAAATCAAGGAGCAT | | | |
| IL1β | CCACCTCAATGGACAGAACATAAG | GACAAACCGCTTTTCCATCTTC | CAAGGAGAGACAAGCAACGACAAAATCCC | | | |
| IL12 | GAAGGCATGGAGCAGGATACAG | TAAATGCAGCACTTCAGAGCCA | CGCTGTGATTCAGAGACCGCATTAGCT | | | |
| IL15 | CATCTATCCAGTTGGCCTCTGTT | TGAGGCTGGCATCCATGTCT | AGGGAGACCTACACTGACACAGCCCAAAA | | | |
| IL4 | TGAACCAGGTCACAGAAAAAGG | ATTCACGGTGCAGCTTCTCA | CAACAAGGAACACCACGGAGAACGAG | | | |
| IL6 | TGTTGTTGACAGCCACTGCCT | CAGTGCATCATCGCTGTTCATA | CTTCACAGAGGATACCACCCACAACAGACC | | | |
| MIP-1a | ATATGGAGCTGACACCCCGACT | CCAGCTCAGTGATGTATTCTTGGA | TCCTGACCAAGAGAAACCGGCAGATC | | | |
| MIP-1b | AGTGCTGTCAGCACCAATAGG | TTTGCCTGCCTTTTTTGGTC | TCCCACTTCCTGCTGCTTCTCTTACACCT | | | |
| MIP-1r | TGTAACCATCCCAATTGCCTC | CGCACTTTATTAGTGGCTTACTGG | CCATTCTGCCTTAGCCCATTAATATGCCTG | | | |
